# The Importance of Terminal Values and Religious Experience of God’s Presence and God’s Absence in the Lives of University Students with Various Levels of Empathy

**DOI:** 10.1007/s10943-014-9884-5

**Published:** 2014-06-17

**Authors:** Stanisław Głaz

**Affiliations:** Jesuit Academy Ignatianum in Krakow, ul. Kopernika 26, 31-501 Kraków, Poland

**Keywords:** Empathy, Religious experience, God’s presence, God’s absence, Students, Young people, Terminal values

## Abstract

The aims of the research I embarked on were: (a) to show the preference of terminal values in personal and in social character, as well to determine the level of religious experience—God’s presence and God’s absence, in groups of young people characterized by a high and low level of empathy and (b) to show the relation between terminal values in personal and in social character and religious experience: God’s presence and God’s absence, in groups of young people with a high and low level of empathy. In the research, the following methods were applied: The Scale of Religious Experience by Głaz—in order to define the level of religious experience: God’s presence and God’s absence, and Mehrabian and Epstein’s *Questionnaire* Measure of Emotional *Empathy*—in order to define the level of empathy. In order to show the terminal values preference amongst young people, the Rokeach Value Survey was applied. The research was carried out in Kraków amongst 200 university students. The research has shown that students with a high level of empathy reveal a higher level of experience of God’s presence than the people with a low level of it. University students with a high level of empathy amongst terminal values prefer most two values in personal character, that is wisdom and pleasure, and one in social character—family security. Similarly, students with a low level of empathy prefer most also two values in personal character, that is pleasure and freedom, and one in social character—family security. In the group of people with a high level of empathy, it is value in personal character—a sense of accomplishment—that contribute more to explaining the variance of religious experience of God’s presence, and in group of people with a low level of empathy, it is social value—social recognition. Whereas in the group of people with a high level of empathy it is value in social character—equality—that contribute more to explaining the variance of religious experience of God’s absence, and in group of people with a low level of empathy, it is personal value—salvation.

## Introduction

Taking into consideration the fact that terminal values—constituting an individual’s most important goals and aspirations—the consequences of religious experience of God’s presence and God’s absence as well as an ability to empathize are all part of human personality (Eisenberg et al. 2004; Głaz 2003; Huber II and MacDonald 2012), it is expected that there are significant relations between them occurring in the lives of university students. On the basis of the theory of those concepts, scientists have created appropriate study tools. The aim of this article is to show the relation between terminal values in personal and in social character and religious experience of God’s presence and God’s absence in the lives of students with a high and low level of empathy.

### Values

A value is commonly conceived of as any aim (Popielski 2008) or object of aspiration for a human being (Schwartz 1992). A value is considered as something that directs human behaviour in a given culture, that is respectable and desirable owing to a certain goal (Opoczyńska 1995). Values perform an important role—namely, they regulate individual and community lives in a given society (Oleś 2003). In this paper, Rokeach’s definition of value was applied. According to Rokeach (1969, 1973), the value concept is a constant belief that a specific mode of conduct or end (ultimate) state of existence is personally and socially superior to an opposite or converse mode of conduct or end state of existence. Rokeach distinguished two kinds of values: terminal, which are in personal and in social character and which define the end state of human desires and aspirations (such as personal freedom, salvation), and instrumental values, which are in moral character and denote competency and which are seen as desirable modes of conduct (e.g. helpfulness, self-control). The terminal values denote aims that people set, whereas the instrumental values are modes of conduct thanks to which those aims can be achieved. Particular values exist within an orderly system. Hence, a human ability to point to values which are higher or lower in his or her hierarchy of values. Rokeach Value Survey (RVS) was used in this paper. The Polish cultural adaptation of it was made by Brzozowski (1986).

### Religious Experience

Research shows great deal of confusion regarding the nature of religious experience. Some researchers describe this experience as profoundly religious. It is perceived as type of genuine and immediate contact with a power recognized as divine presence (Otto 1968) or divine reality (James 1908; Jung 1982). Others show that in origin, it has no religious reference. For instance, it is thought that religious experience could be a result of conflict between id and ego in the human being (Freud 1961a) and that religious experience may be sourced by psychedelic drugs (Smith 1964; Clark 1969; Grof 1995). It is also quite common to identify experiences invoked both by psychedelic substances (boundary experiences) or the brain activity stimulated by means of external tools (neurotheology) with religious experience caused by God’s doing (Newberg 2010). On the one hand, religious experiences can be helpful in explaining the mystery of existence (Fromm 1966; Jung 1982; Frankl 1987; Krok 2009) but, on the other hand, as claimed by Freud (1951, 1961b) and others, it falsely assures of God’s careful providence, which eventually leads to self-deception and neurotic disorders.

On the basis of the theoretical concepts by James (1908), Allport (1950) and Maslow (1962), researchers have created several research tools for studying religious experience. These include: Mysticism Scale (Hood 1975; Hood et al. 1989), Centrality of Religiosity Scale (Huber 2003) and Mystical Experience Questionnaire (MacLean 2012). The analysis of literature by many Christian mystics (Juan de la Cruz, Teresa de Avila, Ignacio de Loyola) reveals that in their lives they experienced God’s presence, His leaving, His being silent, His return (Głaz 1998). The author of this paper, on the basis of the concept of religious experience in accordance with the Christian religion (Rahner 1984; Głaz 1998), created a study tool for measuring the intensity of God’s absence and God’s presence (Scale of Religious Experience—SRE) (Głaz 2011). The following elements should be distinguished in it: the subject of religion (a human being), the object of religion (God personified) and religious relation, which is in personal character. The latter can be defined as a kind of bond with God personified through which a human being attains the aims of his or her own existence. In the process of religious experience, we have shown three stages: the openness of a human being to God, communication of God with a human being and acceptance by the human being of the influence of God’s spirit that inspires, comforts, heals and lives in harmony with themselves and the universe. Religious experience finds realization during a religious act (Jarosz 2003). Religious act is of relational, transcendent, cognitive and evaluative nature (James 1908; Scheler 1980; Radziszewski 2009). A human being, when entering a relation with God, gains new knowledge about another person and herself or himself. Through that knowledge, God ‘speaks’ to them and a human being responds with their faith, love and involvement (Zdybicka 1979). What decides about the type of religious experience are elements brought in by both man and God, taking into consideration the uniqueness and distinctive features of those elements (Głaz 1998). That is why a human being shows two different kinds of religious experience in their life: experience of God’s absence and of God’s presence. These sorts of experience are specific and unique. They are frequently a source of different and new knowledge, which can be subject to psychological analysis. They are also a reason why opposing feelings appear in human life, like happiness—sadness, elation—doubt. The intensity of those feelings, which are set in a human personality, is volatile, and their consequences are visible in a human being’s attitude (Głaz 2011).

### Empathy

Research results so far show a lack of a uniform opinion amongst psychologists about the understanding of empathy (Hoffman 1987; Coplan 2011). Empathy is defined as ‘vicarious introspection’, the ability to know the inner life of another (Kohut 1977). It may be the most powerful factor for personal change (Eisenberg 2002), growth and development of a human being (Rogers 1975). The concept of empathy as understood by Mehrabian and Epstein (1972) was applied. It is perceived as an ability to put yourself in another person’s position. It is an ability to understand another person’s world, their experiences and to feel, experience and understand their emotional reactions, both positive and negative ones. It is revealed, amongst others, by an individual’s emotional empathy to another person’s present mental state and by active responsiveness. According to Mehrabian and Epstein, empathy is one of the many concepts of a human being’s social behaviour and presents an important field for researchers. It encompasses a regulatory function in man which determines various forms of interaction. Interaction, in turn, requires from people being in personal contact to build up mutual understanding. Mehrabian and Epstein (1972), on the basis of theory of empathy, created a test—The Questionnaire Measure of Emotional Empathy (QMEE)—which was used in this paper. The Polish cultural adaptation of it was made by Rembowski (1989).

### Problem and Objective Research

Scientific research has led to some important conclusions: empathy, values and religious experience play a significant role in the process of human development, in personal and social behaviour, and they are also helpful in health and care professions (Schwartz 1992; Głaz 2010; Huber II and MacDonald 2012). People with a high level of empathy are also more in control of their aggressive behaviour, they respect pro-family and moral values more than people characterized by a low level of empathy (Kaźmierczak 2004; Krysińska 2000). Research by Czerniawska (2002) reveals that aggressive youth prefer hedonistic values, whereas young people who are socially well-adapted respect moral-religious values more. People with a high level of empathy respect most terminal values like family security, mature love, mature love and freedom, whereas people with a lower level of empathy respect more hedonistic values and values aimed at success. Voluntary workers and nurses show a higher level of empathic care about others than people working in other professions (Krysińska 2000). In addition, another study shows that the intensity of religious experience correlates strongly with happiness, frequency of religious practice and personal relationships (Drożdż 2003; Soiński 2006). The results obtained by means of the SRE carried out in groups of young people reveal that the same group of students scored high in the subscale of experience of God’s presence and at the same time in the subscale of experience of God’s absence (Głaz 2011). Personal religiosity has a significant relation with conscience sensitivity to moral values (Buksik 2003; Głaz 2013). Students with a high level of openness to experience show a greater sense of the divine. Their belief is more flexible, and they also reveal more curiosity in searching for new experience than students with a low level of it. They show better relations with their friends and families, a greater feeling of personal security, and they also seem to be associated with greater social involvement (Krok 2009). In a group of men, amongst eighteen terminal values, it is salvation that has the strongest relation to experience of God’s presence, whereas in a group of women it is love. When it comes to experience of God’s absence, turned out that a terminal value ‘salvation’ is the most strongly related to it, both in the group of men and women (Głaz 2006).

However, it must be noted that empathic behaviour can be source of disharmony, and even conflicts with another person and the surroundings. This has been indicated by some research (Reykowski 1979; Carozzi et al. 1995), which leads to the conclusion that the influence of the phenomenon of empathy can be accompanied e.g. by an increase in aggression towards an individual, which is in turn a source of empathic stimulation, release of anxiety and an inclination to self-justification by ignoring any signals of another person’s suffering.

Related literature and empirical studies suggest that empathy, which is revealed in understanding another person’s mental–spiritual world and in one’s attitude to another person, terminal values, which determine a person’s most important aims and aspirations of personal and social character, as well as religious experience of God’s presence and God’s absence, which constitutes an essential element of human religiosity, are all significantly related to one another in the lives of young people (Jaworski 2006; Głaz 2007). The question of the aforementioned factors related to self-accomplishment as a person is familiar to psychologists studying existential issues (Frankl 1987; MacLean et al. 2004; Popielski 2008; Głaz 2011). However, there is still room for further research in that field of interest, which would be more detailed in its nature. Empirical data referring to empathy, terminal values and religious experience found in related literature are, however, unambiguous, due to the fact that they largely depend on the specific of the subject group and on the understanding of a given problem adopted in the study procedure.

According to Buksik (2003) and Popielski (2008), on the one hand, it is a person’s sensitivity that decides about the choice of values and their realization, and, on the other hand, the attractiveness of the desired values. The findings of the studies by Czerniawska (2002), Kula-Lic (2008) and Rembowski (1989) suggest that a coherent system of values and their internalization enriched by empathy increase the appearance of a coherent pro-social behaviour as well as young people’s ability to restrain from aggressive behaviour. According to Frankl (1987) and Śliwak (2001), a human being, under the influence of sensitivity to another person’s mental state and their understanding, decides whether to undertake a certain pro-social action or not, and, at the same time, whether a given value will be realized at that moment or not.

In the context of these problems, the following questions arise: To what extent does the level of a human being’s individual trait, which is empathy, as a personal predisposition, modify and define the compliant with it choice of terminal values, which perform a regulatory function in a human life? To what extent does the character of terminal values—understood as personal standards—imply the experience of God’s presence and God’s absence?

By suggesting an existence of a content compliance between a person’s individual traits such as empathy, values in personal and in pro-social character—which perform a regulatory function in a person’s behaviour, define and co-create the style of social relations and the form of interpersonal communication (Jones 2001; Popielski 2008) as well as between the religious experience of God’s presence and God’s absence, it is expected that there is a significant relation between these aspects of human life.

According to Rokeach (1973) and Popielski (2008), everyone has their own hierarchy of values. The most preferred value takes up a central role in his or her life and also performs an essential regulatory function in his or her personality and religious life. It is expected that terminal values most respected by people contribute significantly to explaining the variance of religious experience of God’s presence and God’s absence. According to Rokeach (1973), terminal values in personal character strongly focus on an individual and accomplishment of his or her existence, whereas terminal values in social character concern more the relation and attitude to another person and the surroundings. Hence the expectation is that terminal values in social character have a significant relation to a high level of empathy. Similarly, it is suggested that in the group of people with a high level of empathy it is terminal values in social character, rather than in personal, that have a stronger influence on religious experience: God’s presence and God’s absence.

### Questions and Hypotheses

The aim of the study of this paper is to seek answers to the following study questions:

1. Does the level of empathy differentiate (a) terminal values in personal and in social character (b) and religious experience, that is experience of God’s presence and God’s absence?
2. To what extent and in which order do the above mentioned variables relating to terminal values in personal and in social character explain the variance of dependent variables related to religious experience: experiencing God’s presence and God’s absence, in the groups of people with a high and low level of empathy?

The research problem suggests the following hypotheses:

1. Students characterized by a high level of empathy, amongst terminal values prefer values in social character, whereas students with a low level of empathy prefer values in personal character.
2. There exists a significant relation between the most preferred terminal values by young people with a high level of empathy and religious experience of God’ presence and God’s absence.
3. In the group of students characterized by a high level of empathy, terminal values in social character contribute more to explaining the variance of religious experience of God’s presence, whereas in the group with a low level of empathy—values in personal character.
4. In the group of students characterized by a high level of empathy, values in social character contribute more to explaining the variance of religious experience of God’s absence, whereas in the group with a low level of empathy—values in personal character.

## Methods

### Participants and Procedure

A question appears: who to include as subjects of the study? In times of searching for new solutions, both in private and in social life, in the period of young people’s changing one set of values for another, and the phase of one’s own religious change in Poland (Mariański 2001; Drozdz 2003; Soiński 2010), it is advisable to conduct a study amongst young university students of various faculties. What is typical for early adulthood is that a young person, after turbulent developmental changes leading to social adulthood, is aware of his or her ability to procreate, responsibility to take on new social roles, professional work and autonomy to direct their own life (Rogers 1983; Pervin 1993). Early adulthood is a time when authentic religiosity is formed and differentiating criteria appear, such as religious from non-religious feelings (Fromm 1966; Popielski 2008).

The research was carried out amongst 293 students, who were tested in order to produce empirical data which could be used to find the answers to the questions that had been raised. The study took place amongst university students in 2010 in Kraków. The subject group comes from several non-Catholic public and Polish state universities. All students were Polish born, culturally homogeneous, and stemmed from families of average affluence. All the students declared belonging to the Roman-Catholic Church. The age of the respondents varied between the ages of 21 and 25 (*M* = 23.1; SD = 12.09). Two hundred properly filled questionnaires were taken into account during the analysis (there were two groups: 100 males and 100 females). All questioned persons declared themselves as religious and all of them had religious experience of God’s presence and God’s absence.

### Instruments

In order to solve the research problem, the following tools were applied:

The SRE by Głaz in aim to measuring the intensity of religious experience of God’s presence and God’s absence. It is comprised of a set of 37 statements in religious character and referring to the Christian religion. They take into account the following elements: the object of religion (God personalized), the subject of religion (a human being), and a relation in personal character. For the interpretation of the results of factor analysis, the variables (statements) whose loadings exceeded 0.400 were taken into account. The scale has three sub-scales. The first one describes the intensity of the experience of God in general (DB); Cronbach’s alpha coefficient of internal consistency is 0.92. The second scale serves to measure the intensity of the experience of God’s presence (OB), with Cronbach’s alpha coefficient of internal consistency at 0.94. The third scale describes the intensity of the experience of God’s absence (NB). Cronbach’s alpha coefficient of internal consistency is also high and stands at 0.91 (Głaz 2011). The scale was tested on several samples, which included students of Catholic state universities as well as elderly people. The results obtained are related. The correlation coefficients are at a high level and positive (0.54–0.64). Criterion validity: correlation (p Spearman) between the SRE and Prężyna’s Scale of Religious Attitude is 0.63.

Rokeach Value Survey was applied in order to determine values preference amongst students. It consists of two sub-scales. Each sub-scale consists of 18 values. One of the sub-scales is used to measure the preference of terminal values (personal and social), i. e. the most important goals and desires, whereas the other sub-scale is used to order instrumental values (moral and competence), which are the most general modes of conduct. Rokeach estimated the permanence of each value (test reliability) by the test–retest method (*N* = 250) and the scores for terminal values were coefficients ranging from 0.51 to 0.88, and for instrumental values from 0.45 to 0.70. The ranking correlation coefficients between the Polish and American versions of the terminal and instrumental values scales were 0.99 and 0.98 respectively. The (*r*) Pearson coefficients for individual positions equalled: in case of terminal values—on average 0.79, and for instrumental values—on average 0.68 (Rokeach 1973). In the present paper, only the scale of terminal values in personal and in social character was used.

The Questionnaire Measure of Emotional Empathy (QMEE) by Mehrabian and Epstein was applied in order to measure the level of empathy. It is a method of self-description. Its authors referred to the already existing empathy scales, for instance those by Kerr or Dymond. The authors of the questionnaire, Mehrabian and Epstein (1972), include the emotional and cognitive element. They define empathy as a certain ability to imagine yourself in another person’s position and to understand and experience their emotional reactions, both positive and negative ones. The method consists of thirty-three statements referring to various situations, to which one is to respond according to his or her level of sensitivity. The result is then noted on the 9-point rating scale—from very strong disagreement to very strong agreement. This tool allows us to estimate the respondents’ overall intensity of empathy (Rembowski 1989).

### Statistical Analysis

The variable referring to empathy was dichotomized. Hence, the criterion for division was values achieved in the QMEE. Two groups of people were formed, who scored high or low in the empathy questionnaire. The young people’s low scores on empathy (both males and females) range within ≤4.5, and the high scores ≥5.0. The group with a low level of empathy consists of fifty-four people, the mean (*M*) for the group being 5.81 (SD = 0.971), whereas the group with a high level of empathy consists of sixty-eight people, the mean (*M*) being 5.45 (SD = 0.748). There is a statistically significant difference between the two groups with *F* = 21.13; *p* < 0.001.

The study results amongst university students obtained by means of the SRE and the RVS were further analysed. In order to show the significance of differences between the groups of respondents (with a high and low level of empathy), the analysis of variance (ANOVA) was applied. The results were subsequently interpreted on the basis of the mean values (*M*) and standard deviations (SD). For the variables expressed by ranks, a nonparametric significance test (Mann–Whitney *U* test) was applied. Showing statistically significant differences and rank values will enable to find answers to the main study question—namely, to what extent the lifestyle differentiates the world of terminal values in personal and in social character as well as religious experience: God’s presence and God’s absence. Also, the procedure of the analysis of the multiple stepwise regression was applied. This type of method has several functions: it aims at finding independent variables (so-called ‘significant’); it describes the relation between an independent variable and a dependent variable (multiple regression coefficient—*R*); and it sets the percentage value of the explanatory variance of a dependent variable (*R* ^2^); it also shows the order in which independent variables are entered into the equation of the regression.

## Results

### The Level of Religious Experience of God’s Presence and God’s Absence in Groups of Students with a High and Low Level of Empathy (Table 1)

The results gained in the SRE indicate (Table 1) that there is a statistically significant difference in the factor relating to experience of God’s presence (OB). People with a high level of empathy also reveal a higher level of experience of God’s presence (OB) than the people with a low level of it (*p* < 0.01). However, when it comes to experience of God’s absence (NB), both groups scored similarly achieving an average result, with no significant difference noticeable.

**Table 1:** The analysis of variance (ANOVA), value of the *F* test and the level of significance *p* for the variables in the groups with a high (H) and low (L) level of empathy relating to religious experience of God’s presence (OB) and God’s absence (NB)

Factors	Students with a high level of empathy (H)		Students with a low level of empathy (L)		*F*	*df*	*p*
	*M*	SD	*M*	SD			
OB	5.24	0.978	4.10	1.510	14.06	1	<0.01
NB	3.81	0.757	3.57	0.995	1.45	1	0.67

### Ranking Distribution of Terminal Values in Personal and in Social Character in Groups of Students with a High and Low Level of Empathy (Table 2)

The results achieved in the Rokeach Value Scale (RVS) relating to terminal values (Table 2), taking into consideration the three most preferred values, indicate that people with a high level of empathy seem to prefer values such as wisdom (3.00), pleasure (5.0) and family security (5.0) most. In the group of people with a low level of empathy, the most preferred values are pleasure (6.0), freedom (6.0) and family security (7.0).

**Table 2:** Ranking distribution of terminal values in personal (p) and in social (s) character in groups of university students with a high and low level of empathy

Groups	Students with a high level of empathy (H)	Ranks	Students with a low level of empathy (L)	Ranks
Most preferred terminal values	Wisdom (p)	3.0	Pleasure (p)	6.0
	Pleasure (p)	5.0	Freedom (p)	6.0
	Family security (s)	5.0	Family security (s)	7.0

### Relation of Terminal Values with Religious Experience of God’s Presence in Groups of Students with a High and Low Level of Empathy (Table 3)

Amongst the eighteen terminal values, eight seem to have a significant relation with religious experience of God’s presence (OB). In the group of people with a high (H) level of empathy, there are three terminal values relating to a sense of accomplishment (9t), true friendship (6t) and salvation (16t). The first one, namely a sense of achievement (9t), explains 9 % of the variance of experience of God’s presence (OB) (*R* = 0.3012, and all three values explain 21 % of the variance of that experience (OB) (*R* = 0.4617). Goodness of fit of the multiple stepwise regression equation sets the *F* test at *F* = 4.20; *df* = 3; *p* < 0.05. The multiple stepwise regression equation being OB = 4.91 + 0.08 × 9t + 0.10 × 6t + 0.13 × 16t. In the group of people with a low (L) level of empathy, these are terminal values relating to social recognition (11t), world peace (5t), self-respect (10t), pleasure (7t), wisdom (3t) and inner harmony (12t). The first value, namely social recognition (11t), explains 18 % of the variance of experience of God’s presence (OB) (*R* = 0.4297), and all three together explain 60 % of the variance of that experience (OB) (*R* = 0.7721. Goodness of fit of the multiple stepwise regression equation sets the *F* test at *F* = 9.60; *df* = 6; *p* < 0.05. The multiple stepwise regression equation being as follows: OB = 2.9 + (−0.04) × 11t + 0.09 × 5t + 0.13 × 10t + (−0.10) × 7t + 0.21 × 3t + 0.19 × 12t.

**Table 3:** Independent variables relating to terminal values in personal (p) and in social (s) character explaining the variance of religious experience: God’s presence (OB) in groups of students with a high (H) and low (L) level of empathy

Groups	Values explaining the variance of experience of God’s presence (OB)	*B*	*R*	Percentage of explained variance (*R* ^2^ × 100 %)
H	A sense of accomplishment (9t) (p)	0.08	0.3012	9
	True friendship (6t) (s)	0.10	0.3904	15
	Salvation (16t) (p)	0.13	0.4617	21
L	Social recognition (11t) (s)	−0.04	0.4297	18
	World peace (5t) (s)	0.09	0.5422	29
	Self-respect (10t) (p)	0.13	0.6463	42
	Pleasure (7t) (p)	−0.10	0.7015	49
	Wisdom (3t) (p)	0.21	0.7394	55
	Inner harmony (12t) (p)	0.19	0.7721	60

The results of the multiple stepwise regression analysis

### Relation of Terminal Values with Religious Experience of God’s Absence in Groups of Students with a High and Low Level of Empathy (Table 4)

Only three terminal values amongst eighteen, such as equality (8t), salvation (16t) and mature love (2t) bear a relation to religious experience of God’s absence (NB). In the group of people with a high (H) level of empathy, it is the terminal value relating to equality (8t) and it explains merely 6 % of the variance of experience of God’s absence (NB) (*R* = 0.2521). Goodness of fit of the stepwise regression equation sets the *F* test value at *F* = 4.96; *df* = 1; *p* < 0.05. The equation of multiple stepwise regression is as follows: NB = 4.18 + (−0.05) × 8t. In the group of people with a low (L) level of empathy, these are values referring to salvation (16t) and mature love (2t). The first terminal value, namely salvation (16t), explains 17 % of the variance of experience of God’s absence (NB) (*R* = 0.4147), and both values together explain 25 % of the variance of that experience (NB) (*R* = 0.5054). Goodness of fit of the stepwise regression equation sets the *F* test value at *F* = 5.15; *df* = 2; *p* < 0.05. The equation of multiple stepwise regression is as follows: NB = 5.15 + 0.05 × 16t + (−0.09) × 2t.

**Table 4:** Independent variables relating to terminal values in personal (p) and in social (s) character explaining the variance of religious experience of God’s absence (NB) in the groups of students with a high (H) and low (L) level of empathy

Groups	Values explaining the variance of experience of God’s absence (NB)	*B*	*R*	Percentage of explained variance (*R* ^2^ × 100)
H	Equality (8t) (s)	−0.5	0.2521	6
L	Salvation (16t) (p)	0.05	0.4147	17
	Mature love (2t) (s)	−0.09	0.5054	25

The results of the multiple stepwise regression analysis

## Discussion of the Results

The present analysis concerning the relation between terminal values in personal and in social character, which define the most important goals and aspirations of an individual, and religious experience of God’s presence and God’s absence in groups of university students characterized by a high or low level of empathy reveals that there is a significant relation between the aforementioned factors. As a reminder, in the analysis of the terminal values hierarchy, only the three top values were considered which were most preferred by the students in the groups with a high and low level of empathy.

The first hypothesis, which suggests that students with a high level of empathy amongst terminal values prefer values in social character most, and students with a low level of empathy prefer values in personal character, was only partly confirmed. University students with a high level of empathy amongst terminal values prefer most two values in personal character, that is wisdom and pleasure, and one in social character—family security. It was expected—in accordance with other research (Czerniawska 2002)—that in the group of students with a high level of empathy there would be more terminal values in social character. Similarly to students with a high level of empathy, students with a low level of it prefer most two terminal values in personal character and one in social character. They refer to pleasure, freedom and family security. It was expected that young people with a low level of empathy prefer values in personal character more, which can be implied from related literature (Rembowski 1989).

The second hypothesis, which suggests that the most respected terminal values have a significant relation to religious experience of God’s presence and God’s absence in the group of people wit a high level of empathy, was not fully confirmed. None of the most respected terminal values have a significant relation to the process of appearance of religious experience of God’s presence and God’s absence in the group of people wit a high level of empathy. However, two terminal values in personal character (wisdom, pleasure) named as most respected have a significant relation to experience of God’s absence in the group of people with a low level of empathy. It seems that the treatment of the construct of emotional empathy cannot receive a one-dimensional consideration. In this case—in accordance with some researchers’ views (Eisenberg 2002; Eisenberg et al. 2004)—these could well be other emotional elements of empathy such as co-feeling or expressing one’s feelings. Rulla (1997) and Eisenberg (1986) also appear to be right pointing out that controversial research results on the relation between the phenomenon of empathy and behaviour or some people’s acting are often determined by adopting by researchers not fully developed forms of empathy, which sometimes can co-exist with other immature aspects of personality.

The first part of the third hypothesis, which indicates that in the group of students with a high level of empathy it is terminal values in social character that contribute more to explaining the variance of religious experience of God’s presence, was only partly confirmed. Namely, amongst eighteen terminal values in the group of young people with a high level of empathy, two terminal values in personal character (a sense of accomplishment, salvation) and one in social character (true friendship) have a significant relation to experience of God’s presence and explain its variance. The strongest terminal value in this configuration turned out to be a value in personal character—a sense of accomplishment. Whereas the second part of this hypothesis, which suggests that in the group of people with a low level of empathy it is terminal values in personal character that have an influence on experience of God’s presence, also was only partly confirmed. The four terminal values which entered the regression equation and explain the variance of experience of God’s presence in this group of young people are all in personal character (self-respect, pleasure, wisdom, inner harmony), and only two in social character (social recognition, world peace).

The four hypothesis, which indicates that in the group of young people with a high level of empathy it is values in social character that contribute more to explaining the variance of religious experience of God’s absence, and in the group of people with a low level of empathy it is personal values, was not fully confirmed. In the group of young people with a high level of empathy, only one terminal value, in social character—equality, has a significant relation with experience of God’s absence and explains its variance. However, two terminal values—salvation (in personal character) and mature love (in social character)—have a significant relation to experience of God’s absence and explain its variance. It was expected, in accordance with the second part of the fourth hypothesis, that in the group of university students with a low level of empathy it is terminal values in personal character that have a significant relation to experience of God’s absence.

It was expected that level of empathy, as a human being’s personality predisposition, differentiates religious experience of God’s presence and God’s absence. Subject literature clearly suggests such a relation (Allport 1950; Soiński 2006), whereas the present analysis failed to confirm fully its existence. Young people with a high level of empathy have a higher level of experience of God’s presence than people with a low level of empathy. Both groups of students, however, with a high and a low level of empathy, reveal equal level of religious experience of God’s absence. It suggests that these two elements of human life—religious experience of God’s presence and empathy—are to a certain extent content dependent. Empathy and religious experience of God’s absence, though, seem to have different psychological backgrounds, are shaped by other factors (e.g. uncertainty) and are content independent.

The results obtained reveal that young people characterized by a high or low level of empathy respect values and have their own hierarchy of values which constitutes an important part of their personalities and religious life. According to Rokeach (1973) and Popielski (2008), a central part in a human being’s life is filled with the value he or she respects most, which proves its significance and an important regulatory function in his of her personal and religious life. In the lives of university students characterized by a high and low level of empathy, a central place is taken by terminal values in personal character. It means that, regardless of the level of empathy, terminal values in personal character, which evolve around an individual and accomplishment of his or her existence, perform a significant regulatory function in the lives of university students. It concerns values such as wisdom and pleasure in the group of people with a high level of empathy and pleasure and freedom in the group with a low level of empathy. The value ‘pleasure’ performs a regulatory function both in the group of young people with a high and with a low level of empathy. The third most preferred terminal value, and at the same time one that has an important position in the values hierarchy of the young people, both in the group characterized by a high and low level of empathy, turned out to be a value in social character—family security.

It was expected, in accordance with other research findings (Czerniawska 2002; Batson et al. 2004) and subject literature (Davis 1999), that students with a high level of empathy amongst terminal values respect more values in social character, whereas students with a low level of empathy respect values in personal character most, which the present study failed to confirm, though. The obtained results suggest that in this case the preferred terminal values, as standards of young people’s strives, are compliant with the definition of an empathic personality only to a certain extent (Davis 1983, 1999), which seems to prove a content incongruity in their certain dimensions between these two aspects of human life.

The strongest value amongst the eighteen terminal values turned out to be a value in personal character—salvation, which focuses on an individual and concerns the accomplishment of his or her existence. It has a significant relation with experience of God’s presence in the group of students with a high level of empathy and with experience of God’s absence in the group of students with a low level of empathy. It seems to indicate—which finds its confirmation in other research (Głaz 2007) that that value substantially contributes to creation of religious experience of both God’s absence and God’s presence, and it also performs an important regulatory function in young people’s personal and religious lives.

Amongst eighteen terminal values, seven in personal and two in social character have a significant relation to experience of God’s presence in the group of students with a high level of empathy. It seems to suggest, according to Popielski (2008), that religious experience of God’s presence, as one of the elements of a human being’s religiosity, is more determined by terminal values in personal character, which means the ones more individual oriented and which refer to self-perfection, than values in social character, which means the ones which define interpersonal relations in one’s environment.

It was not expected that the most terminal values (six) have a significant relation with experience of God’s presence in the group of young people with a low level of empathy and it is them that explain the most of the variance of that experience (60 %). It suggests that the contribution of a high number of terminal values as well as their strong input in the appearance of experience of God’s presence in the lives of students with a low level of empathy can be caused by the fact that in the lives of students in this case terminal values as personal standards, their attractiveness and usefulness, perform a greater regulatory function in the religious life of students and imply their experience of God’s presence than empathic sensitivity.

It was also expected that more terminal values—either personal or social—have a significant relation to experience of God’s absence both in the group of students with a high and low level of empathy. It turned out that only one terminal value—equality—has a significant relation to God’s absence in the group of students with a high level of empathy and it explains merely 6 % of the variance of that experience. Researchers tend to find a strong relation between empathy and religiosity; however, in this case that relation is hard to be found.

Researchers (Oleś 2003; Oliynichuk and Popielski 2008) seem to be right in noticing that it is not known to what extent the preferred values are only respected, desired and to what extent they are actually applied in the lives of young people. One has to admit, though, that the hierarchy of values presented by university students and its significant relation to religious experience of God’s presence and God’s absence as well as to empathy suggest that terminal values perform a significant regulatory function in their personal and religious lives. It concerns especially terminal values in personal character, which are related to the accomplishment of one’s existence.

The present analysis of the subject matter shows a significant relation between terminal values in personal and in social character, which point to young people’s most important goals and aims and which perform an important regulatory function in their personal and religious life, and religious experience of God’s presence and God’s absence, as well as with the level of empathy amongst university students. All those dimensions considered in the present analysis are autonomous in their cognitive, emotional, evaluative and aspiring relations; nevertheless, they form a unity within human reality. They decide about students’ spiritual and mental development as well as the way they experience existence.

The results obtained suggest that there must exist other personality aspects which have a significant relation to experience of God’s presence and God’s absence; certain variables that considerably contribute to the process of religiosity and personality formation. This process is especially active in one’s youth. And these are the aspects that need to be revealed in future studies.
